# Evaluation of PepT1 (SLC15A1) Substrate Characteristics of Therapeutic Cyclic Peptides

**DOI:** 10.3390/pharmaceutics14081610

**Published:** 2022-08-01

**Authors:** Gzona Bajraktari-Sylejmani, Teresa von Linde, Jürgen Burhenne, Walter Emil Haefeli, Max Sauter, Johanna Weiss

**Affiliations:** Department of Clinical Pharmacology and Pharmacoepidemiology, University of Heidelberg, Im Neuenheimer Feld 410, 69120 Heidelberg, Germany; gzona.bajraktari-sylejmani@med.uni-heidelberg.de (G.B.-S.); t.linde@stud.uni-heidelberg.de (T.v.L.); juergen.burhenne@med.uni-heidelberg.de (J.B.); walter.emil.haefeli@med.uni-heidelberg.de (W.E.H.); max.sauter@med.uni-heidelberg.de (M.S.)

**Keywords:** PepT1, substrate, pasireotide, octreotide, glycyl-proline, glycyl-sarcosine, LC–MS/MS, CHO-K1

## Abstract

The human peptide transporter hPepT1 (SLC15A1), physiologically transporting dipeptides and tripeptides generated during food digestion, also plays a role in the uptake of small bioactive peptides and peptide-like drugs. Moreover, it might be addressed in prodrug strategies of poorly absorbed drugs. We hypothesised that the cyclic drug peptides octreotide and pasireotide could be substrates of this transporter because their diameter can resemble the size of dipeptides or tripeptides due to their strong structural curvature and because they reach the systemic circulation in Beagle dogs. For investigating possible hPepT1 substrate characteristics, we generated and characterised a CHO-K1 cell line overexpressing *SLC15A1* by transfection and selection via magnetic beads. Possible inhibition of the uptake of the prototypical substrate Gly-Sar by octreotide and pasireotide was screened, followed by quantifying the uptake of the cyclic peptides in cells overexpressing *SLC15A1* compared with the parental cell line. Although inhibition of Gly-Sar uptake was observed, uptake of octreotide and pasireotide was not increased in *SLC15A1* overexpressing cells, indicating a lack of transport by hPepT1. Our data clearly indicate that octreotide and pasireotide are nonsubstrate inhibitors of hPepT1 and that their oral bioavailability cannot be explained by absorption via hPepT1.

## 1. Introduction

The human peptide transporter PepT1 (SLC15A1) physiologically mediates the uptake of dietary dipeptides and tripeptides by cotranslocating protons along the proton gradient at the brush border membrane of the small intestine [1,2,3]. Apart from its high abundance in the intestine, PepT1 is also expressed in the renal proximal tubulus and the epithelium of the bile duct [2,3]. PepT1 is a pH-dependent high-capacity but low-affinity transporter and exhibits a high substrate promiscuity, transports nearly all physiological dipeptides and tripeptides, and consequently plays an important role in the nutritional supply with amino acids [2,3]. Further, it also mediates the uptake of several peptide-like drugs, such as angiotensin-converting enzyme or protease inhibitors and β-lactam antibiotics [3].

The development of cyclic peptides as drugs is of special interest. Compared with linear peptides they have higher metabolic stability, and compared with small-molecule drugs, they can interact with larger surface areas of the target protein, which makes them more specific and even allows inhibition of protein–protein interactions that cannot be influenced by small molecules [4]. However, a crucial shortcoming of most cyclic peptides is their impermeability to membranes, resulting in low bioavailability and limiting their efficacy against intracellular targets. Therefore, understanding the mechanisms that allow cyclic drugs to penetrate cell membranes is of extraordinary importance for oral formulation development.

**Figure 1:**
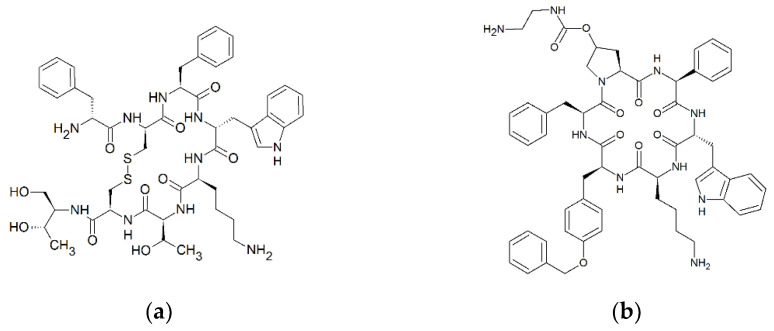
Cyclic structure of octreotide (D-Phe-cyclo[Cys-Phe-D-Trp-Lys-Thr-Cys]-Thr; C_49_H_66_N_10_O_10_S_2_) (**a**) and pasireotide (cyclo[Hyp(N-(2-aminoethyl)carbamate)-Phg-D-Trp-Lys-Tyr(Bn)-Phe ; C_58_H_66_N_10_O_9_) (**b**). Formulas were drawn with ChemSketch 2020.1.2. (ACD/Labs, Toronto, ON, Canada).

The two approved cyclic peptide therapeutics octreotide and pasireotide are synthetic somatostatin analogues and, therefore, somatostatin receptor agonists. Octreotide is especially applied in the treatment of growth-hormone-secreting tumors, such as acromegaly [5], and pasireotide is primarily used for the treatment of Morbus Cushing [6]. During an evaluation of the oral pharmacokinetics of cyclic peptide analogues of somatostatin in Beagle dogs, we observed that octreotide and pasireotide are absorbed to a considerable extent (bioavailability of >1%, Sauter et al., unpublished data), indicating a transport across the intestinal barrier. In addition, experiments with enantiomeric pairs of cyclic hexapeptides have indicated an enantiotopic-dependent, carrier-mediated transport of polar cyclic peptides across Caco-2 cell monolayers [7]. Based on these observations, we hypothesised that small cyclic peptides might exhibit substrate characteristics for PepT1 because their diameter can resemble the size of dipeptides or tripeptides due to their strong structural curvature (ring structure; the polar surface area of octreotide (332 Å) and pasireotide (281 Å) [8] are comparable to that of tripeptides, such as GWG (265 Å) [9]) (Figure 1), and because PepT1 shows an enantiotopic substrate preference (L-amino acid peptides over D-amino acid peptides) [2]. If this can be confirmed, the oral absorption of cyclic peptide therapeutics may be modulated with (chemical) modifications that enhance PepT1 binding and, as a consequence, may facilitate the rational development of oral peptide formulations. 

Therefore, we evaluated whether the small cyclic peptide somatostatin receptor agonists (≤8 amino acids) octreotide and pasireotide are substrates of hPepT1. For this purpose, we generated and characterised an hPepT1 overexpressing Chinese hamster ovarian K1 (CHO-K1) cell line. After characterisation, the clone with the highest expression was used for further experiments. First, we screened whether the cyclic peptides inhibit the Gly-Sar uptake in a semi-high-throughput assay to obtain a first indication of an interaction with hPepT1. For peptides inhibiting hPepT1, we assessed their uptake in the cell line overexpressing hPepT1 compared with the parental cell line CHO-K1. 

## 2. Materials and Methods

### 2.1. Materials

Octreotide acetate and pasireotide trifluoroacetate were purchased from Bachem AG (Bubendorf, Switzerland). Hank’s buffered salt solution (HBSS), phosphate-buffered saline (PBS), penicillin, streptomycin sulfate, Gly-Sar, Gly-Pro, 2-(N-morpholino)ethanesulfonic acid (MES), low protein binding microtubes, and the GenElute™ Mammalian Total RNA Miniprep Kit were obtained from Sigma-Aldrich (Taufkirchen, Germany). Cell culture medium Ham’s F12, foetal calf serum (FCS), and DMEM were purchased from PAN-Biotech (Aidenbach, Germany). Dimethyl sulfoxide (DMSO), aprotinin, pepstatin A, Triton X-100, geneticin (G418), and bovine serum albumin (BSA) were from AppliChem (Darmstadt, Germany). Leupeptin was obtained from Biomol (Hamburg, Germany), and Pefabloc from Carl Roth (Karlsruhe, Germany). The MS columns plus tubes and the anti-PE microbeads were obtained from Miltenyi (Bergisch Gladbach, Germany). Losartan potassium and the monoclonal anti-SLC15A1 antibody E-3 PE (SC-373742) were purchased from Santa Cruz Biotechnology (Heidelberg, Germany). The RevertAid™ H Minus First Strand cDNA Synthesis Kit, the RIPA lysis and extraction buffer, and the Pierce™ BCA protein assay kit were purchased from Thermo Fisher Scientific (Waltham, MA, USA). The QuantiTect Hs_SLC15A1 primer set and the QuantiFast SYBR Green Mix were obtained from Qiagen (Hilden, Germany). Primers for the reference genes were synthesised by Eurofins MWG Operon (Ebersberg, Germany). The mammalian expression vector pcDNA3.1+/C-(K)DYK–SLC15A1 (ABIN4935376) was obtained from genomics-online (Aachen, Germany). The FuGENE^®^ HD transfection reagent was from Promega (Madison, WI, USA). [^13^C3]-sarcosine was obtained from Toronto Research Chemicals (North York, ON, Canada). Ammonia solution (25%), N,N-dimethylformamide (DMF), and trifluoroacetic acid (TFA) were obtained from Merck (Darmstadt, Germany). Tert-butyl methyl ether (TBME) was provided by VWR International (Darmstadt, Germany). Purified water was produced using an Arium^®^ Mini (Sartorius, Göttingen, Germany) ultrapure water system. The remaining reagents and solvents, methanol (MeOH), acetonitrile (ACN), and formic acid (FA) were purchased from Biosolve (Valkenswaard, the Netherlands) in the highest purity available.

### 2.2. Stock Solutions

A 50 mM stock solution of MES (2-(N-morpholino)ethanesulfonic acid) contained 50 mM MES, 136.9 mM NaCl, 5.4 mM KCl, 1.26 mM CaCl_2_, 0.81 mM MgSO_4_ × 7 H_2_O, 0.5 mM Na_2_HPO_4_, 0.44 mM KH_2_PO_4_, 0.42 mM NaHCO_3_, and 5.54 mM glucose. The pH value was adjusted to pH = 6.0. Stock solutions of Gly-Sar, Gly-Pro, and losartan were prepared in MES buffer and stored in aliquots at 4 °C.

### 2.3. Cell Culture

Chinese hamster ovarian cells (CHO-K1) were obtained from DSMZ–German Collection of Microorganisms and Cell Cultures GmbH (Braunschweig, Germany) and cultured in DMEM/Ham’s F12 with 10% FCS, 100 U/mL penicillin, and 100 µg/mL streptomycin sulfate under standard cell culture conditions. The medium of the transfected cell lines after selection additionally contained 500 µg/mL G418.

### 2.4. Transfection

CHO-K1 cells were seeded on 6-well plates at a concentration of 2.25 × 10^6^ cells/well, leading to 60–80% confluency on the day of transfection. One day after seeding, the complete medium was replaced by medium without FCS, and cells were starved for 4 h. Afterwards, cells were transfected in complete medium with a ratio of 1.5 × FuGENE to 1 × pcDNA3.1+/C-(K)DYK–SLC15A1 according to the manufacturer’s instructions. Before further proceeding, cells were cultivated for 48 h under standard cell culture conditions. 

### 2.5. Selection of Stable CHO-hPepT1 Cell Lines via Cloning Cylinders and G418 Treatment

After 2 d of recovery, medium was replaced and cells in different wells were treated with different concentrations of G418 (100 µg/mL, 200 µg/mL, or 400 µg/µL). After 7 d of culture, single clones were selected via cloning cylinders, trypsinised, and transferred to single wells of 24-well plates. Ten days after selection of the clones, the G418 concentration was set to 400 µg/mL and further increased to 500 µg/mL 17 d after selection.

### 2.6. Isolation of Transfected CHO-K1 Cells via Magnetic Bead Isolation

For bead isolation, cells were detached from the 6-well plates by trypsinisation after being treated for 5 min with 5 mM EDTA solution to decrease the trypsinisation time. Afterwards, cells were washed with PBS, and 2.5 × 10^6^ cells were resuspended in 800 µL PBS/0.5% BSA. After adding 20 µL of the PE-labelled anti-SLC15A1 antibody E-3, cells were incubated for 15 min on ice. Following washing with ice-cold PBS, cells positive for PepT1 and thus binding the PE-labelled antibody were isolated via anti-PE microbeads according to the manufacturer’s instructions. Isolated cells were grown in complete cell culture medium containing 200 µg/mL G418 and after the first passage switched to complete medium with a final concentration of 500 µg/mL G418.

### 2.7. Quantification of SLC15A1 mRNA Expression

RNA was reverse-transcribed to cDNA with the RevertAid™ H Minus First Strand cDNA Synthesis Kit using oligo-dt primers according to the manufacturer’s instructions. mRNA expression was quantified by real-time reverse transcription (RT) polymerase chain reaction (PCR) with LightCycler^®^ 480 (Roche Applied Science, Mannheim, Germany) as described previously [10]. For quantifying *SLC15A1* mRNA, the QuantiTect Hs_SLC15A1 primer set was used. The primers for the human *SLC15A1* also detected the homologous gene of the hamster, thus enabling us to also quantify the basal expression of PepT1. As reference genes for normalisation, methylmalonic aciduria and homocystinuria type D protein (*Mmadhc*) and peptidyl-prolyl cis-trans isomerase (*Fkbp1A*) were used, because they were identified to be stably expressed genes in CHO cells [11]. Primers were published previously [11]. PCR amplification was carried out in 20 µL reaction volume containing 5 µL 1:10 diluted cDNA, 1× QuantiFast SYBR Green Mix and 1× QuantiTect Hs_SCL15A1 or 0.15 µM sense and antisense primers each. Data were evaluated via calibrator-normalised relative quantification with efficiency correction using the LightCycler^®^ 480 software version 1.5.1.62 (Roche Applied Science, Mannheim, Germany). Results were expressed as the target/reference ratio divided by the target/reference ratio of the calibrator. Thus, the results were corrected for variance caused by detection and sample inhomogeneities. All samples were amplified in technical duplicate.

### 2.8. Validation of hPepT1 Protein Functionality in the Clones CHO-hPepT1-M5 and CHO-hPepT1-200 via Gly-Sar Uptake and Its Inhibition

The functionality of the overexpressed hPepT1 transporter was verified by measuring the uptake of the prototypical substrate Gly-Sar. For each sample, 5 × 10^5^ cells were suspended in 1.5 mL low-binding reaction tubes and washed once in MES buffer. Subsequently, cells were incubated in 250 µL of the respective Gly-Sar solution (20 µM up to 20 mM) at 37 °C for 10 min on a rotary shaker (450 rpm). Cells were then washed twice with 0.5 mL ice-cold MES buffer, and the pellet was lysed in 50 µL aqueous NH_4_OH (10%). Quantification of Gly-Sar via UPLC–MS/MS was conducted and validated as described previously [12]. Cell volume was measured with a CASY Cell Counter (OLS Omni Life Science GmbH, Bremen, Germany), and results were calculated as intracellular concentrations. Each Gly-Sar concentration was tested in triplicate, and the experiment was conducted thrice.

In a second step, inhibition of hPepT1 by losartan, one of the strongest inhibitors of this transporter [10], was tested in both clones. Inhibition experiments were conducted as previously described at a Gly-Sar concentration of 20 µM [13]. In brief, cells were seeded in 96-well plates 1 d before the assay at a concentration of 5 × 10^4^ per well. On the day of the inhibition experiment, cells were washed once with MES buffer (100 µL/well) and subsequently incubated with 20 µM Gly-Sar and losartan (0.1–5000 µM), all prepared in MES buffer. After an incubation period of 10 min at 37 °C on a rotary shaker (450 rpm), transport was terminated by removing the solution and washing the cells with ice-cold MES buffer thrice while the plate was kept on ice. Afterwards, cells were lysed in the 96-well cell culture plate with aqueous NH_4_OH (10%, 50 µL/well), and sample preparation was performed as described previously [13]. Inhibition in the clone CHO-hPepT1-M5 was also tested with Gly-Pro (0.1–5000 µM) as described for losartan.

### 2.9. Inhibition of the Gly-Sar Uptake in CHO-hPepT1-M5 Cells

The cyclic peptides were first evaluated for hPepT1 inhibition with the semi-high-throughput assay in 96-well plates established and validated earlier [13], but using CHO-hPepT1-M5 instead of Caco-2 cells. Cells were seeded 2 d before the assay at a concentration of 5 × 10^4^/well, and the assay was conducted as published previously [13]. Each concentration was tested in octuplet, and each experiment performed thrice.

### 2.10. Uptake of Cyclic Peptides in CHO-hPepT1-M5 Cells

For investigating the possible hPepT1 substrate characteristics of the cyclic peptides, their uptake was measured in the CHO-hPepT1-M5 clone with and without inhibition by Gly-Pro and compared with the CHO-K1 parental clone.

For each sample, 5 × 10^5^ cells were suspended in 1.5 mL low-binding reaction tubes and washed once in MES buffer. Subsequently, cells were incubated in 100 µL of the respective cyclic peptide solution in MES with or without 20 mM Gly-Pro at 37 °C for 10 min on a rotary shaker (450 rpm). Cells were then washed twice with 0.4 mL ice-cold MES buffer before further proceeding as described in Section 2.8. Each concentration was tested in triplicate, and the experiment was conducted at least thrice. Only cells within passages 1–5 after thawing were used for these experiments, and overexpression of *SLC15A1* was confirmed via PCR after each passage.

### 2.11. Intracellular Quantification of Cyclic Peptides by UPLC–MS/MS

To quantify intracellular concentrations, previously established UPLC–MS/MS methods for the bioanalysis of octreotide [14] and pasireotide [15] were adapted for an ultrasensitive quantification in cell lysates. Cell lysis was performed with 50 µL of 10% aqueous ammonia, and the lysates acidified with 50 µL of 20% aqueous FA to ensure stability of the peptide therapeutics during sample processing. Sample preparation and UPLC–MS/MS measurements were performed as previously described for plasma [12,13]. The assays were revalidated for cell lysates with regard to accuracy, precision, and linearity and fully complied with the required limits (accuracy within 100 ± 15% and precision ≤ 15%) of the recommendations for bioanalytical method development of the FDA and EMA [16,17].

### 2.12. Statistical Analysis

Nonlinear regression curves were calculated with GraphPad Prism version 9.3.1 (GraphPad Software Inc., La Jolla, CA, USA) using the four-parameter fit (sigmoidal dose-response curves with variable slope) for inhibition experiments of a one-phase association nonlinear curve fit for the Gly-Sar uptake. For testing differences in the uptake of octreotide and pasireotide in CHO-K1 and CHO-hPepT1-M5 cells, a two-way ANOVA followed by a Bonferroni post hoc test was used. A *p*-value < 0.05 was considered significant. 

## 3. Results

### 3.1. Generation of hPepT1 Overexpressing Cell Lines

After transfection, cell clones were selected either via cloning cylinders and treatment with G418 or alternatively via magnetic bead isolation. Altogether, 21 clones were isolated and maintained at a G418 concentration of 500 µg/µL after several passages. The expression of *SLC15A1* mRNA was then quantified by quantitative real-time RT-PCR.

**Figure 2:**
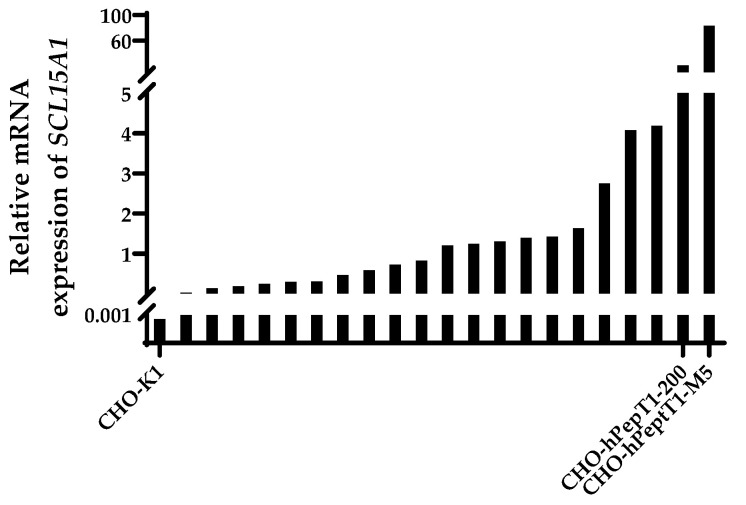
Relative mRNA expression of *SLC15A1* in CHO-K1 and the CHO-K1 clones after transfection with *SLC15A1* and selection. Data were normalised to the reference genes *Fkbp1a* and *Mmadhc*. Depicted is the mean of a technical duplicate. The two clones with the highest expression (CHO-hPepT1-200 and CHO-hPepT1-M5) were chosen for further characterisation.

All transfected and selected clones exhibited an increase in the mRNA expression of *SLC15A1* compared with the parental cell line (Figure 2). The clones with the highest relative mRNA expression (CHO-hPepT1-200 and CHO-hPepT1-M5) were selected for further characterisation by testing whether the overexpressed transporter was indeed functional. 

### 3.2. Functional Characterisation of the Clones CHO-hPepT1-M5 and CHO-hPepT1-200

To verify the suitability of the two selected clones for identifying hPepT1 substrates, the uptake characteristics of the prototypical substrate Gly-Sar were assessed in these cell lines compared with the parental cell line CHO-K1.

**Figure 3:**
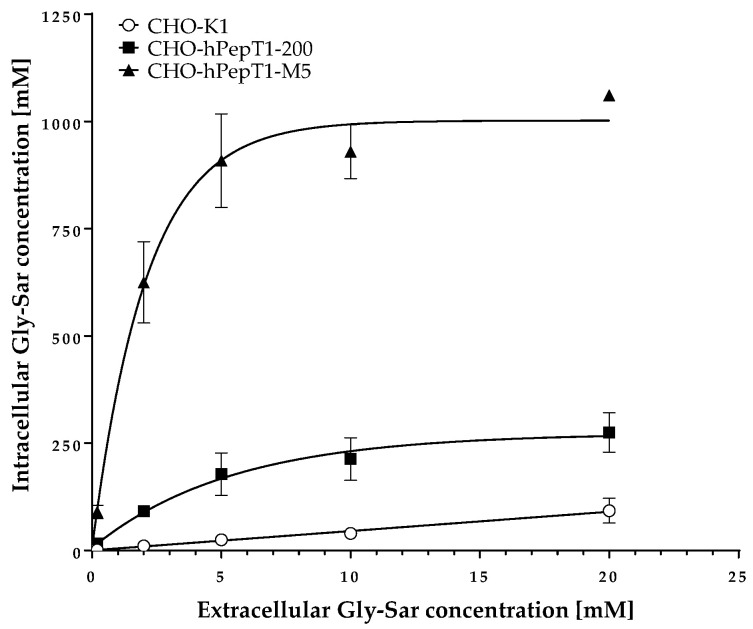
Uptake of Gly-Sar in CHO-K1, CHO-hPepT1-200, and CHO-hPepT1-M5 clones. The data points represent the mean ± S.E.M. of three independent experiments with each concentration tested in triplets.

**Figure 4:**
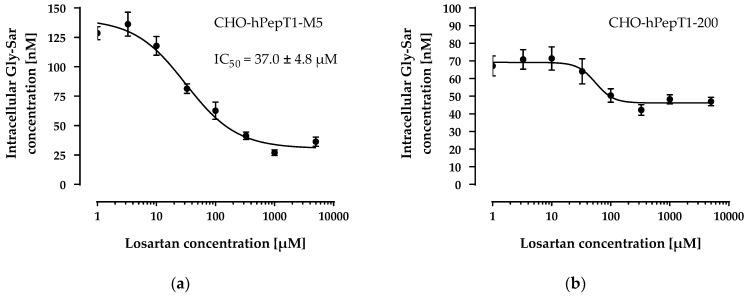
Concentration-dependent inhibition of the Gly-Sar uptake in the CHO-hPepT1-M5 (**a**) and the CHO-hPepT1-200 (**b**) clone by losartan. The data points represent the mean ± S.E.M. of three independent experiments with each concentration tested in sextuplets.

The data presented in Figure 3 clearly demonstrates a concentration-dependent and saturable uptake of Gly-Sar in the two overexpressing cell lines. In contrast, in the parental cell line, the uptake of Gly-Sar was much lower and not saturable up to 20 mM. Thus, the higher mRNA expression of *SLC15A1* in the CHO-hPepT1-M5 cells compared with the CHO-hPepT1-200 cells (about five times higher, Figure 1) is also reflected by a higher functional activity in this cell clone. To verify whether the uptake of Gly-Sar can really be attributed to PepT1, its inhibition was tested by using losartan and Gly-Pro, two well-known inhibitors of this transporter. Figure 4a demonstrates a typical concentration-dependent inhibition of the Gly-Sar uptake in CHO-hPepT1-M5 cells by losartan (IC_50_ = 37.0 ± 4.8 µM), indicating that the Gly-Sar uptake can primarily be attributed to functional PepT1. In contrast, in CHO-hPepT1-200 cells, the inhibition curve was much flatter (Figure 4b), indicating that the inhibition range was too small to propagate this cell clone as suitable for the detection of a PepT1 substrate or inhibitors.

**Figure 5:**
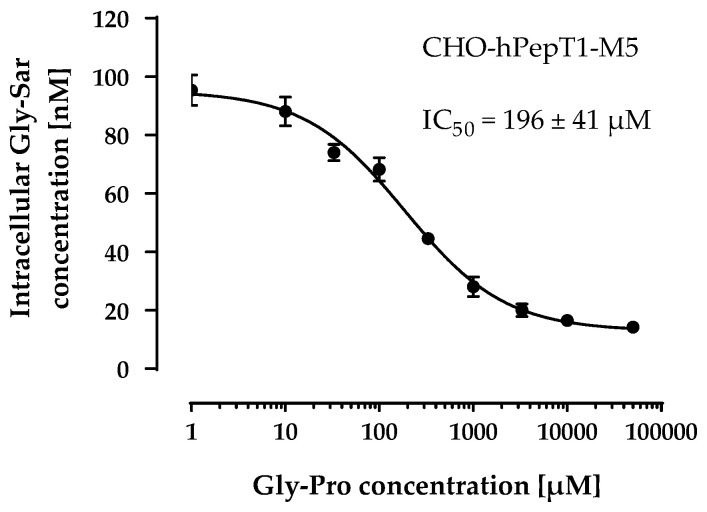
Concentration-dependent inhibition of the Gly-Sar uptake in the CHO-hPepT1-M5 clone by Gly-Pro. The data points represent the mean ± S.E.M. of three independent experiments with each concentration tested in sextuplets.

Therefore, we used the M5 clone for all subsequent experiments and verified its PepT1 activity via inhibition with a second inhibitor (Gly-Pro). As observed with losartan, Gly-Pro inhibited the Gly-Sar uptake in CHO-hPepT1-M5 cells concentration-dependently (IC_50_ = 196 ± 41 µM; Figure 5) and confirmed that the M5 clone expresses functional hPepT1.

### 3.3. Inhibition of Gly-Sar Uptake in CHO-hPepT1-M5 Cells by Octreotide and Pasireotide

**Figure 6:**
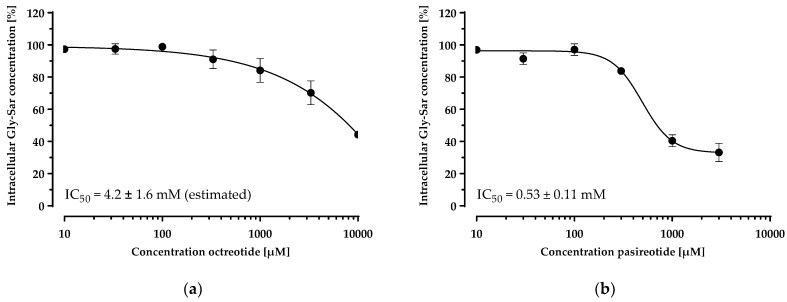
Concentration-dependent inhibition of the Gly-Sar uptake in the CHO-hPepT1-M5 clone by octreotide (**a**) and pasireotide (**b**). The data points represent the mean ± S.E.M. of three independent experiments with each concentration tested in octuplets.

Having established and characterised a PepT1 overexpressing cell line, we investigated the inhibition of the Gly-Sar uptake by the cyclic peptide drugs in this cell line (Figure 6). Both peptides inhibited Gly-Sar uptake concentration-dependently. The disulfide-cyclised octapeptide octreotide showed an IC_50_ of 4.2 ± 1.6 mM for the inhibition of Gly-Sar uptake (estimated due to the incomplete sigmoidal concentration–response curve). The head-to-tail cyclised hexapeptide pasireotide inhibited Gly-Sar uptake with an IC_50_ of 0.53 ± 0.11 mM. 

### 3.4. Uptake of Octreotide and Pasireotide in CHO-hPepT1-M5 Cells

**Figure 7:**
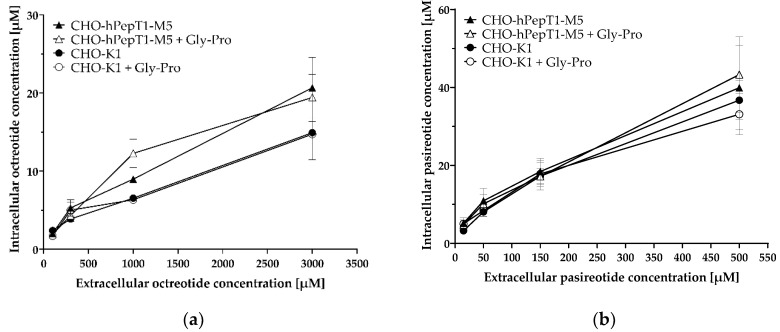
Uptake of octreotide (**a**) and pasireotide (**b**) in CHO-hPepT1-M5 cells and CHO-K1 cells with and without the inhibitor Gly-Pro. Cells were incubated for 10 min with the respective peptides. Each data point represents the mean ± S.E.M. for three experiments with each concentration tested in triplicate.

The inhibition experiments demonstrated a concentration-dependent decrease in the Gly-Sar uptake by octreotide and pasireotide and thus indicated an inhibition of PepT1 by the two cyclic peptides. To investigate whether octreotide and pasireotide are also substrates of hPepT1, we investigated their uptake in the *SCL15A1* overexpressing cell line compared with the parental cell line and in absence and presence of the PepT1 inhibitor Gly-Pro (Figure 7).

The data presented in Figure 7 demonstrate no obvious difference in the uptake of octreotide and pasireotide in cells without (CHO-K1) or cells with hPepT1 overexpression (CHO-hPepT1-M5) as tested by a two-way ANOVA, followed by a Bonferroni post hoc test. Accordingly, the PepT1 inhibitor Gly-Pro had no significant influence on the uptake of these peptides. As expected, there was a significant concentration-dependent increase in intracellular accumulation in all treatments (*p* < 0.0001).

## 4. Discussion

Cyclic peptides attract increasing interest as therapeutics due to their ability to inhibit proteins or protein–protein interactions that cannot be addressed with small molecules. Their application, however, is restricted by their low membrane permeability. In general, several possible mechanisms have been proposed on how cyclic peptides enter cells. Among them are (1) passive diffusion, (2) endocytosis, (3) direct translocation, and (4) active transport [4]. 

For some cyclic hexapeptides, a carrier-mediated transport has been proposed [7], and we observed a respectable bioavailability of octreotide and pasireotide in beagle dogs of >1% (Sauter et al., unpublished results), also suggesting that there might be active transport mechanisms for cyclic peptides. Because the diameter of small cyclic peptides can resemble the size of dipeptides or tripeptides due to their strong structural curvature, we hypothesised that cyclic peptides may show substrate characteristics for PepT1. We therefore investigated possible PepT1 substrate characteristics of the cyclic peptides octreotide and pasireotide, for which no uptake mechanism has yet been identified.

For testing PepT1 inhibition, we recently established a semi-high-throughput screening method using Gly-Sar as a substrate and Caco-2 cells as a cellular model for PepT1 activity [13]. This cell line is quite suitable for investigating the inhibition of PepT1, because it expresses considerable amounts of this transporter [18], and when using a suitable substrate, such as Gly-Sar, the assay quite specifically detects PepT1 inhibitors. However, Caco-2 cells express a multitude of transporters, making them less adequate for investigating substrate characteristics. We therefore decided to establish a cell model overexpressing *SLC15A1* and used the easily transfectable and often used CHO-K1 cells as the starting cell line. We successfully generated several clones with *SCL15A1* overexpression using two different selection methodologies, antibiotic resistance selection and isolation with specific magnetic beads. The clone with the highest expression was obtained via magnetic bead isolation (CHO-hPepT1-M5). This clone also demonstrated favorable functional hPepT1 characteristics and was thus used for the inhibition and uptake assays with the cyclic peptides. 

Although octreotide and pasireotide inhibited Gly-Sar uptake in CHO-hPepT1-M5 cells (Figure 6), these peptides do not appear to be hPepT1 substrates, because their uptake into hPepT1 overexpressing cells did not differ from that of the parental cell line and the hPepT1 inhibitor Gly-Pro had no influence on their uptake (Figure 7). The peptide solution in MES buffer had a pH of 6, which proves that a pH deviation cannot be the cause of the observed inhibition characteristics. For PepT1, there are also several described nontransported inhibitors, such as losartan [19]. Considering the fact that typical PepT1 substrates are dipeptides or tripeptides, octreotide and pasireotide might simply be too large to be transported, even if they can bind to PepT1. Moreover, an indirect inhibition of Gly-Sar transport might occur by the breakdown of the proton gradient by unspecific effects on the cell membrane [19], or by direct interaction of Gly-Sar with the cyclic peptides (e.g., by ion-pair formation of the acid function of Gly-Sar with amino functions of the cyclic peptides). However, pasireotide showed an almost 10-fold higher inhibition efficacy compared with octreotide, questioning a relevant role of proton gradient disruption or ion-pair formation with Gly-Sar by the peptides themselves. Because pasireotide has a much stronger structural curvature than octreotide due to an amino acid chain that is two amino acids shorter and the head-to-tail cyclisation and therefore a considerably smaller polar surface area, pasireotide may fit (and bind) the hPepT1 binding site substantially better, which could explain its lower IC_50_ value. Furthermore, the basicity of pasireotide and octreotide is very similar due to the identical presence of basic functions (two amino functions and one tryptophan each) without acidic moieties, and therefore, indirect inhibition by the breakdown of the proton gradient by the peptides should result in similar IC_50_ values. Therefore, consistent with our hypothesis, especially pasireotide likely competes for binding to PepT1, inhibiting Gly-Sar uptake without being transported.

Taken together, hPepT1 does not appear to be responsible for the uptake of octreotide and pasireotide, bringing other uptake transporters into focus. Some cyclic peptides have been demonstrated to be transported by members of the organic anion transporting polypeptide family (OATPs), such as the bicyclic peptides demethylphalloidin by OATP1B1 and OATP1B3 [20] and the bicyclic amanitin by OATP1B3 [21]. Therefore, in future studies, it seems worth investigating whether octreotide and pasireotide are also transported by OATPs.

Limitations: (1) We did not generate a mock control for the CHO-hPepT1-M5 cell line but used the parental cell line as a control because the vector without the insert coding for *SLC15A1* was not available. However, the functional data obtained with the Gly-Sar uptake in the M5 clone compared with the parental cell line clearly indicate the suitability of the overexpressing clone. (2) Expression of hamster PepT1/PepT2 in the CHO cells might have influenced our results. This especially applies to PepT2, which is, in contrast to PepT1, a high-affinity and low-capacity transporter. However, so far, there are no convincing data that one of these transporters is expressed in CHO-K1 cells in relevant amounts. Next-generation sequencing of CHO-K1 cells even failed to detect the expression of hamster PepT2 at all [22]. Moreover, the linear nonsaturable uptake of Gly-Sar (Figure 3) does not indicate relevant active transport of Gly-Sar in CHO-K1 parental cells excluding the substantial contribution of hamster PepT1 and PepT2.

## 5. Conclusions

We established an *SLC15A1* overexpressing CHO-K1 cell line suitable to investigate possible hPepT1 inhibitor and substrate characteristics. The cyclic peptide therapeutics octreotide and pasireotide inhibited the Gly-Sar uptake in this cell line, but our data indicate that they are not transported by hPepT1.

## Data Availability

Data are available from the corresponding author upon reasonable request.
